# A Case of Pernicious Anemia Presenting With Severe Hemolysis

**DOI:** 10.7759/cureus.50534

**Published:** 2023-12-14

**Authors:** Kaitlyn N Romero, Falguni Patel, Oshin Rai, Austin Quan, Pramod Reddy

**Affiliations:** 1 Internal Medicine, University of Florida College of Medicine – Jacksonville, Jacksonville, USA

**Keywords:** intrinsic factor, megaloblastic anemia, pernicious anemia, hemolytic anemia, vitamin b12 deficiency anemia

## Abstract

Vitamin B12 deficiency is a well-known and overall common disease. While the etiology of vitamin B12 deficiency varies from post-surgical changes to inadequate dietary consumption, pernicious anemia should be considered as it is a common cause. Pernicious anemia is an autoimmune atrophic gastritis impairing the absorption of vitamin B12. Manifestations include neurological changes, macrocytic anemia, glossitis, and nail changes. Hemolytic anemia is an unusual complication of vitamin B12 deficiency and an even more unusual initial presentation. This case identifies a patient with previously undiagnosed pernicious anemia with severe vitamin B12 deficiency compounded by hemolytic anemia as the presenting symptom. Overall, this case highlights the importance of considering vitamin B12 deficiency-related hemolytic anemia and the need for further research into the causes and pathophysiology of vitamin B12-induced hemolysis due to its potential for fatal outcomes despite being easily treatable with cost-effective methods to treat.

## Introduction

Pernicious anemia is an autoimmune disease characterized by vitamin B12 deficiency due to antibodies that target intrinsic factor (IF) or parietal cells, both directly involved in vitamin B12 absorption [1]. In the United States, vitamin B12 deficiency affects about 20% of individuals over 60 years old; however, the prevalence is significantly decreased in patients younger than 60 years old with only 6% affected [2]. Patients with pernicious anemia can be asymptomatic for up to 10 to 20 years prior to presentation, which can manifest as neuropsychiatric and even hematological symptoms. More commonly, patients present with macrocytic anemia in addition to fatigue, shortness of breath, and pallor [1,2]. Without supplementation of vitamin B12, neuropsychiatric conditions develop such as subacute combined degeneration, bilateral and symmetrical paresthesias, loss of vibratory and positional sense, and memory and mood disturbances [1]. Pancytopenia and hemolysis are rare presentations of pernicious anemia, especially in developed nations such as the United States [3]. In this case, we present a middle-aged female with severe anemia in the setting of newly discovered pernicious anemia with laboratory findings indicating hemolysis in which prompt repletion of vitamin B12 resolved said hemolysis.

## Case presentation

A 39-year-old female with a past medical history of laparoscopic cholecystectomy in 2021 presented with complaints of a progressive, one-month history of generalized weakness, periorbital tingling, and lightheadedness. The patient stated symptoms had been constant but exacerbated by overexertion. Her menstrual cycle was described as regular and light with no clots. She has a single 5 cm uterine fibroid stable on ultrasound last month. The patient stated she eats red meat, dairy products, and vegetables daily, denying a vegetarian or vegan diet. Social history was negative for alcohol, tobacco, and illicit drug use. The patient denied any prescribed medications or supplements aside from a daily multivitamin. Family history was negative, including colon cancer or any gastrointestinal malignancies. Review of systems was negative overall including symptoms of active bleeding, B-type symptoms, bowel changes, syncope, skin rash, myalgias, oral ulcers, and arthralgias.

At presentation, the patient was hemodynamically stable and in no acute distress with vital signs within normal limits except for sinus tachycardia at 110 beats per minute, blood pressure of 115/69 mmHg, and BMI of 27. The physical examination was significant for mild subconjunctival pallor and capillary refill greater than three seconds. There were no skin or nail findings visualized, koilonychia, glossitis, gait abnormalities, or focal neurological deficits.

**Figure 1:**
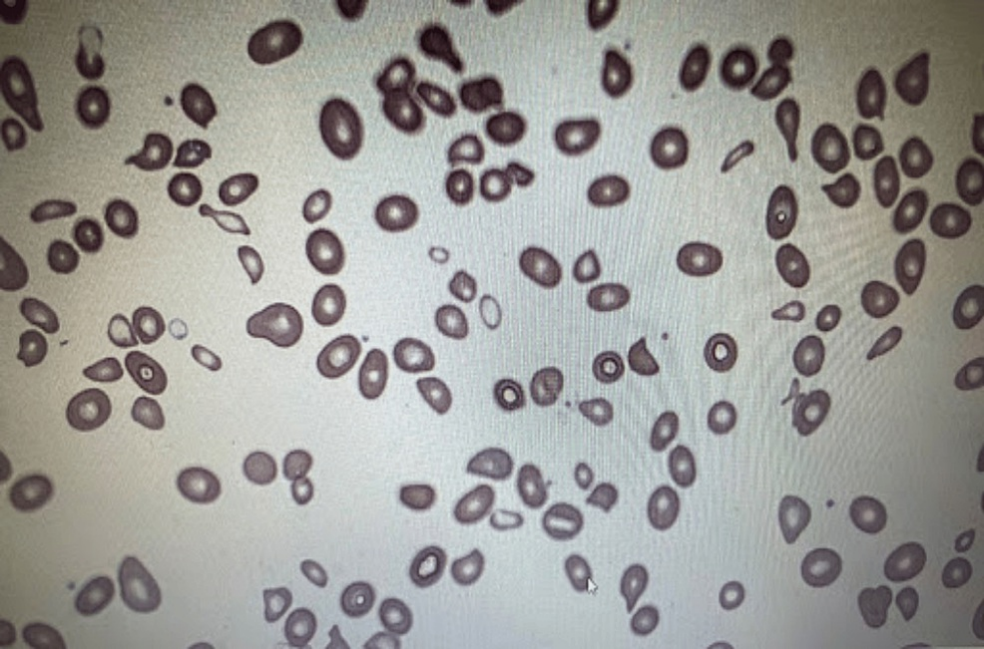
Peripheral blood smear demonstrating macrocytes with schistocytes

Initial laboratory workup was significant for severe macrocytic anemia (hemoglobin 5 g/dL, mean corpuscular volume 113 fl) with mild transaminitis (AST 129 IU/L) and hyperbilirubinemia (total bilirubin 1.3 mg/dL). A decreased haptoglobin level (<10mg/dL) and elevated lactic dehydrogenase result (3,450 IU/L) raised concern for hemolytic anemia (Table 1). Reticulocyte percentage was elevated which indicated an appropriate bone marrow response with potential for hemolysis. Peripheral blood smear showed oval macrocytes with minimal schistocytes (Figure 1). Given the presentation of severe anemia with hemolysis, anemia workup continued and a vitamin B12 level resulted in very low (<150 pg/mL) with homocysteine elevated at 72.6 umol/L (normal range < 14.5 umol/L) and methylmalonic acid (MMA) elevated at 4.2 umol/mmol (normal range < 0.4 umol/mmol). Autoimmune and thyroid workups were unremarkable. Due to a lack of other etiologies from the patient’s history, pernicious anemia was suspected; therefore, intrinsic factor-blocking antibodies (IFBA) were ordered. Serum IFBA was elevated, 248 AU/mL (normal range < 1.1 AU/mL), as were anti-parietal cell antibodies, confirming the diagnosis of pernicious anemia. Gastrointestinal blood loss was ruled out via history and with an adequate iron panel.

**Table 1:** Laboratory findings for a 39-year-old female being evaluated for anemia

	Reference ranges	Admission labs
Ferritin (ng/ml)	12-150 (adult female)	162
Serum iron (ug/dL)	50-170 (adult female)	123
Iron saturation (%)	15-50	46%
Total iron binding capacity (mcg/dl)	250-450	267
Transferrin (mg/dL)	215-380	210
Vitamin B12 (pg/mL)	200-900	<150
Vitamin D25 OH (ng/mL)	20-40	16.8
White cell count (thou/mm3)	4,500-11,000	3.14
Red blood cell (x10e6/uL)	4.2-5.4 (adult female)	1.38
Hemoglobin (g/dL)	12.1-15.1 (adult female)	5
Hematocrit (%)	36-48 (adult female)	15.7
Mean corpuscular volume (FL)	80-100	113.8
Platelet count (x10e3/uL)	150-450	102
Reticulocyte count %	0.5-2.5	3.2
Absolute reticulocyte count (10*6/mm^3^)	0.0225-0.0945	0.0452
Reticulated hemoglobin (PG)	28-36	39.1
Haptoglobin (mg/dL)	41-165	<10
Lactate dehydrogenase (IU/L)	105-333	3,450
Bilirubin, total (mg/dL)	0.1-1.2	1.3
Homocysteine umol/L	4-15	72.7
Intrinsic factor antibodies, serum (AU/mL)	1.21-1.52	248.5

Given severe hemolytic anemia from vitamin B12 deficiency, the patient was administered two units of packed red blood cells with correction of hemoglobin to 8.5 g/dL. The patient was started on intramuscular (IM) injections of 1000 mcg of vitamin B12 daily with a plan to continue for seven days and then transition to oral supplements. The hemoglobin the day after remained stable and even improved to 9.8 g/dL. Given positive initial hemolysis labs without the presence of schistocytes, a direct antiglobulin test (DAT) was planned to further assess hemolysis but was not collected per patient refusal. With vitamin B12 repletion, the hemoglobin improved to 11.7 g/dL and the patient reported resolution of initial symptoms.

## Discussion

The most common causes of vitamin B12 deficiency include gastrointestinal surgery (gastric bypass, ileal resection) and dietary insufficiency and less commonly from autoimmune pernicious anemia [3]. Less commonly, nitrous oxide can cause acute depletion of vitamin B12, where the majority of etiologies are chronic in nature [4]. Vitamin B12 deficiency can result in demyelination of the dorsal and lateral columns of the spinal cord causing subacute combined degeneration as evidenced by paresthesia, sensory deficits, ataxia, and weakness [3]. In patients with known vitamin B12 deficiency, common findings include anemia (37%), macrocytosis (54%), and hypersegmented neutrophils (32%); however, pancytopenia is a rare presentation and is only seen in 5% of patients [5]. Vitamin B12 is used in deoxyribonucleic acid (DNA) synthesis and is required for proper division of hematopoietic cells. Decreased levels of vitamin B12 lead to insufficient DNA replication in the setting of normal cytoplasmic/cell content replication preventing effective cell division resulting in macrocytosis [4]. This lack of maturation and arrest of development of hematopoietic cells may lead to intramedullary cell death resulting in lactate dehydrogenase being released and decreased haptoglobin as seen in our patient [6]. In cases where vitamin B12 or folate deficiency is suspected, it is important to rule out folate deficiency as treating vitamin B12 deficiency with folate can result in improved anemia with worsening neurologic symptoms [4]. MMA levels provide utility in diagnosis, such as in pregnancy when vitamin B12 levels are falsely low in the third trimester or elevated in patients with myelodysplastic syndrome [1,4]. However, limitations of MMA include end-stage renal disease, which can present with elevated MMA levels not accurately indicative of the severity of vitamin B12 deficiency [1]. As with our patient, pernicious anemia should still be considered with a lack of dietary and surgical causes in the patient’s history. 

With pernicious anemia, it is important to start treatment early. Although the anemia typically resolves in four to six weeks, neurologic symptoms can take several months to improve and in some cases be permanent [5]. Treatment of patients with pernicious anemia involves IM injections of vitamin B12 for improved bioavailability [7]. Injection of 1000 mcg of vitamin B12 is to be administered daily for one week followed by weekly injections for four total weeks, then followed by lifelong monthly supplementation with IM injections [8]. Alternatively, regular oral vitamin B12 supplementation (1000-2000 mcg) has been shown to be effective in the treatment of patients with etiologies of megaloblastic anemia [8,9]. A case series demonstrated variability in adequate dosing (500-2000 mcg) seen by lowering of MMA levels and vitamin B12 testing in patients with different etiologies of malabsorption, such as gastrectomy or pernicious anemia [8]. While oral supplementation in these patients may seem ineffective due to a lack of IFs, absorption is believed to be due to IF-independent mechanisms that provide adequate absorption at high enough doses [10]. Overall, the currently accepted treatment of pernicious anemia involves IM injections, with phosphate supplementation adequate for patients that can be closely monitored and followed up.

This case highlights an unusual, yet potentially fatal, aspect of pernicious anemia: hemolysis. Vitamin B12 is required for the conversion of homocysteine to tetrahydrofolate, which is important for DNA production [11]. With B12 deficiencies, accumulation of homocysteine causes oxidative stress to erythrocytes leading to hemolysis as seen in vitro studies; however, the completed pathogenesis is unknown [12]. Overall, the presentation of hemolysis in vitamin B12 deficiencies is rare, consisting of 1.5% of presentations [3]. It is important to consider other causes of hemolysis in patients, such as autoimmune and genetic disorders. In this case, the patient had a negative family history, subacute timing of symptoms occurring close to the fifth decade of life, and symptoms consistent with anemia that resolved with vitamin B12 supplementation, making other etiologies less likely as causes for hemolysis. More often than not, vitamin B12 deficiency hemolytic anemia often presents as non-immune with a negative DAT. In the case above, the DAT was unable to be collected; however, DAT should be collected in the setting of hemolysis, as in pernicious anemia which is an autoimmune process. There are minimal case reports with this presentation, such as a case report in Croatia that reported megaloblastic and autoimmune hemolytic anemia [13]. 

## Conclusions

The case presents an unusual combination of megaloblastic pernicious anemia and hemolytic anemia in the setting of severe vitamin B12 deficiency. The prompt correction of hemoglobin and maintenance of hemoglobin with vitamin B12 administration strengthened our diagnosis. The importance of early recognition of hemolysis in vitamin B12 deficiency can save health costs by decreasing unnecessary workup for other etiologies of hemolytic anemia. Additionally, more research is required to delineate the type of hemolysis (immune versus non-immune), especially in the setting of autoimmune diseases like pernicious anemia. Given this, it is important to test for vitamin B12 deficiency in hemolytic anemia as it is easily corrected.

## References

[1] Socha DS, DeSouza SI, Flagg A, Sekeres M, Rogers HJ (2020). Severe megaloblastic anemia: vitamin deficiency and other causes. Cleve Clin J Med.

[2] Langan RC, Goodbred AJ (2017). Vitamin B12 deficiency: recognition and management. Am Fam Physician.

[3] Andrès E, Affenberger S, Zimmer J (2006). Current hematological findings in cobalamin deficiency. A study of 201 consecutive patients with documented cobalamin deficiency. Clin Lab Haematol.

[4] Moore CA, Adil A (2023). Macrocytic anemia. StatPearls [Internet].

[5] Pelling MM, Kimura ST, Han EJ, Shin YM (2022). Severe vitamin B12 deficiency presenting as pancytopenia, hemolytic anemia, and paresthesia: could your B12 be any lower?. Cureus.

[6] Dalsania CJ, Khemka V, Shum M, Devereux L, Lachant NA (2008). A sheep in wolf's clothing. Am J Med.

[7] Carmel R (2008). How I treat cobalamin (vitamin B12) deficiency. Blood.

[8] Stabler SP (2013). Clinical practice. Vitamin B12 deficiency. N Engl J Med.

[9] Berlin H, Berlin R, Brante G (1968). Oral treatment of pernicious anemia with high doses of vitamin B12 without intrinsic factor. Acta Med Scand.

[10] Doscherholmen A, Hagen PS (1957). A dual mechanism of vitamin B12 plasma absorption. J Clin Invest.

[11] Sadagopan N (2022). Severe hemolytic anemia due to vitamin B12 deficiency in six months. Hematol Rep.

[12] Ventura P, Panini R, Tremosini S, Salvioli G (2004). A role for homocysteine increase in haemolysis of megaloblastic anaemias due to vitamin B(12) and folate deficiency: results from an in vitro experience. Biochim Biophys Acta.

[13] Vucelić V, Stancić V, Ledinsky M (2008). Combined megaloblastic and immunohemolytic anemia associated - a case report. Acta Clin Croat.

